# A *Conus regularis* Conotoxin with a Novel Eight-Cysteine Framework Inhibits Ca_V_2.2 Channels and Displays an Anti-Nociceptive Activity

**DOI:** 10.3390/md11041188

**Published:** 2013-04-08

**Authors:** Johanna Bernáldez, Sergio A. Román-González, Oscar Martínez, Samanta Jiménez, Oscar Vivas, Isabel Arenas, Gerardo Corzo, Roberto Arreguín, David E. García, Lourival D. Possani, Alexei Licea

**Affiliations:** 1 Molecular Immunology and Biotoxins Laboratory, Marine Biotechnology Department, Scientific Research and High Education Center from Ensenada (CICESE), Carretera Ensenada-Tijuana #3918, Zona Playitas, Ensenada 22860, Mexico; E-Mails: jbernald@cicese.edu.mx (J.B.); omgalvan13@yahoo.com.mx (O.M.); mjimenez@cicese.edu.mx (S.J.); 2 Chemistry Biomacromolecules Department, Chemistry Institute, National Autonomous University of Mexico, Av. Universidad 3000, Ciudad Universitaria, PO BOX 70-213, D.F. 04510, Mexico; E-Mails: arg291178@yahoo.com (S.A.R.-G.); arrespin@unam.mx (R.A.); 3 Physiology Department, Medicine Faculty, National Autonomous University of Mexico, Av. Universidad 3000, Ciudad Universitaria, PO BOX 70-250, D.F. 04510, Mexico; E-Mails: oscarlvivasr@msn.com (O.V.); arenas_isabel17@hotmail.com (I.A.); erasmo@unam.mx (D.E.G.); 4 Department of Molecular Medicine and Bioprocesses, National Autonomous University of Mexico, Av. Universidad 2001, C.P. 510-3, Cuernavaca 61500, Mexico; E-Mails: corzo@ibt.unam.mx (G.C.); possani@ibt.unam.mx (L.D.P.)

**Keywords:** *Conus regularis*, nociceptive, calcium channel, eight-cysteine toxin and conotoxins

## Abstract

A novel peptide, RsXXIVA, was isolated from the venom duct of *Conus regularis*, a worm-hunting species collected in the Sea of Cortez, México. Its primary structure was determined by mass spectrometry and confirmed by automated Edman degradation. This conotoxin contains 40 amino acids and exhibits a novel arrangement of eight cysteine residues (C-C-C-C-CC-CC). Surprisingly, two loops of the novel peptide are highly identical to the amino acids sequence of ω-MVIIA. The total length and disulfide pairing of both peptides are quite different, although the two most important residues for the described function of ω-MVIIA (Lys2 and Tyr13) are also present in the peptide reported here. Electrophysiological analysis using superior cervical ganglion (SCG) neurons indicates that RsXXIVA inhibits Ca_V_2.2 channel current in a dose-dependent manner with an EC_50_ of 2.8 μM, whose effect is partially reversed after washing. Furthermore, RsXXIVA was tested in hot-plate assays to measure the potential anti-nociceptive effect to an acute thermal stimulus, showing an analgesic effect in acute thermal pain at 30 and 45 min post-injection. Also, the toxin shows an anti-nociceptive effect in a formalin chronic pain test. However, the low affinity for Ca_V_2.2 suggests that the primary target of the peptide could be different from that of ω-MVIIA.

## 1. Introduction

Conotoxins from cone snails are interesting molecules with a diverse human therapeutic potential, such as anti-nociceptive, antiepileptic, cardio- and neuro-protective activity [1]. They have also become useful tools for research into cancer, neuromuscular and psychiatric disorders [2]. These peptides are potent and highly selective blockers or modulators of ion channel function involved in such disorders. The assessment of the genus *Conus* is the largest single genus of venomous animals known, with around 700 species; considering the fact that each species could express between 100 and 200 venom peptides, it has been estimated that the number of different peptides that can be expressed is at least 70,000 [3]. This could be translated to a surprising amount of different molecules that have been or will be discovered for different molecular targets. 

The structural diversity of such peptides is exhibited at different levels. In addition to highly diverse sequences, these peptides also have a large array of post-translational modifications and highly different cysteine frameworks and disulfide linkages [4]. Among many intriguing features of conotoxins, the cysteine patterns are of special interest, because they are conserved within the conotoxin families and most important, they define the three-dimensional structure of the native peptide. To date, 23 cysteine frameworks have been identified [5]. In this paper, we propose a new cysteine family framework, which should correspond to the number XXIV, according to the conotoxin family nomenclature [6,7,8]. 

The largest and most extensively characterized group of conotoxin peptides that block calcium channels are the ω-conotoxins. The family members of this group contain from 24 to 27 amino acid residues crosslinked by the same type of disulfide arrangements. Usually, they show three particular intramolecular disulfide bounds, which also are known as the four-loop Cys scaffold [9]. They are found in the venom of piscivorous (fish hunters), vermivorous (worm hunters) and molluscivorous (mollusk hunters) cone snails. The most extensively analyzed ω-conotoxin to date is ω-MVIIA, which blocks Ca_V_2.2 ion channels. This conotoxin has been approved by the FDA as a non-opioid analgesic peptide against long-term neuropathic pain in human, under the commercial name of Prialt [10].

However, a non-classical ω-conotoxin has been demonstrated to have activity on calcium ion channels. This newly reported peptide toxin does not have any similarity on primary structure with conventional ω-conotoxins [11]. Therefore, this opens the possibility that not only ω-conotoxins may interact with calcium ion channels.

In the present study, we report the biochemical and functional characterization of the first *Conus regularis* conotoxin (RsXXIVA) isolated from the venom duct. RsXXIVA shows novel eight-Cys patterns and in addition, a section of its primary structure is highly identical to the residues forming two loops of ω-MVIIA. RsXXIVA was tested on rat superior cervical ganglion (SCG) neurons, where it inhibited Ca_V_2.2 calcium currents. Furthermore, it also showed an analgesic effect on mice by using the hot-plate and formalin tests.

## 2. Materials and Methods

### 2.1. Specimen Collection and Venom Extraction

The venom of *Conus regularis* was extracted from the venom duct of 20 specimens collected on the coastal region of the Sea of Cortez, México. It was homogenized in 1 mL of an aqueous solution of 0.1% trifluoroacetic acid (TFA), defined here as solution A. The homogenate was centrifuged at 10,000× *g* for 5 min at room temperature. After centrifugation, the supernatant was separated, lyophilized and stored at −20 °C for further experiments.

### 2.2. Chemicals, Solvents and Materials

In the sample preparation, all solvents (HPLC grade), chemicals and proteins were purchased from Sigma-Aldrich (St. Louis, MO, USA) and used as supplied, unless otherwise stated. ZipTips with C_18_ resin were purchased from Millipore (Millipore, Bedford, MA, USA). 

### 2.3. Peptide Purification

The soluble venom was separated by means of reversed-phase high-performance liquid chromatography (RP-HPLC) using an analytical C_18_ column (Vydac 218TP54; 4.6 × 250 mm, 5 μm particle size). Samples were loaded with solution A, and the venom components were eluted with a linear gradient from 0% to 60% of solution B (0.12% TFA in acetonitrile) at a flow rate of 1 mL min^−1^. Major protein fractions were selected and further separated in a second HPLC step to obtain pure peptides. In particular, the peptide RsXXIVA was obtained at the elution time of 20 min using a micro bore C_18_ column (1.0 × 250 mm, 5 μm) with a linear gradient from 10% to 30% of solution B, at a flow rate of 200 μL min^−1^. In all cases, separation procedures were conducted at room temperature over 60 min, and the absorbance was monitored at 230 nm. 

### 2.4. Amino Acid Sequencing

The primary structure of RsXXIVA was determined by mass spectrometry and confirmed by automated Edman degradation. All mass spectrometry-collision-induced dissociation-ion mobility-mass spectrometry (MS-CID-IM-MS) experiments were performed using a SYNAPT G2 high definition mass spectrometer (HDMS) equipped with a nanoelectrospray ion source and a MassLynx data processor (Waters Corp., Milford, MA, USA). The instrument acquisition parameters used were as follows: an inlet capillary voltage of 1.85 kV, a sampling cone setting of 40 V and a source temperature of 100 °C. The argon pressure in the traveling wave ion guide trap (TWIG-trap) and the traveling wave ion guide transfer (TWIG-transfer) were 2.44 × 10^−2^ and 2.61 × 10^−2^ mbar, respectively. The wave height, the wave velocity and the nitrogen pressure in the traveling wave (TW) IM drift cell were 32.0 V, 850 m/s and 2.96 mbar, respectively. Samples were directly infused into the mass spectrometer at a rate of 0.5–0.8 μL/min. All IM-MS data were acquired in a period of 2 min. All fragmentation was carried out by collision of ions with argon. The collision energy for CID was optimized for each peptide and charge state. The trap collision voltage generally falls in a range from 23 to 50 V. Amino acid sequencing was performed with an automatic gas-phase protein sequencer (LF-3400D TriCart with high sensitivity chemistry; Beckman Coulter, Fullerton, USA). 

### 2.5. Data Analysis

All data processing was conducted using the software, Waters MassLynx v4.1 and DriftScope v2.1. The ion spectra were manually interpreted. To expedite manual interpretation and sequence assignment of mass spectra, an arbitrary cutoff threshold of 10% relative abundance was used in peak assignment for MS-CID-MS experiments, as well as for extracted product ion spectra from MS-CID-IM-MS experiments. Internal fragment ions with a S/N > 3 and an isotope cluster were assigned based on theoretical peak lists generated from the Protein Prospector MS-Product software (UCSF, San Francisco, CA, USA). Internal calibration based on b- and y-type ion masses, as well as an external calibration of the instrument, was utilized to accurately assign all mass/charges. 

### 2.6. Culture of SCG Neurons

SCG neurons were enzymatically dissociated from 5-week male rats (Wistar). Animals were used in accordance with the procedures approved by the Official Mexican Norm NOM 0062-ZOO-1999-entitled technical specifications for the production, care and use of laboratory animals. After dissection, ganglia were desheathed, cut into 8 to 10 small pieces and transferred to a modified Hanks solution containing 20 U/mL of papain. After 20 min at 37 °C, the solution containing papain was replaced with a new solution containing 1 mg/mL of collagenase type I and 10 mg/mL dispase. Ganglia were incubated for 40 min in this solution and mechanically dissociated every 20 min. Later, the preparation was centrifuged and resuspended twice in Leibovitz’s l-15 medium and once in Dulbecco’s modified Eagle’s medium, both supplemented with 10% (v/v) heat-inactivated fetal bovine serum and 1% penicillin–streptomycin. Cells were plated on polystyrene culture dishes coated with poly-l-lysine and stored in a humidified atmosphere containing 5% CO_2_ in air at 37 °C. Neurons were studied between 15 and 24 h after plating. All recordings were obtained at room temperature (19 to 22 °C).

### 2.7. Electrophysiological Recording

Neurons were constantly perfused during recording (1–2 mL/min) with a solution designed to isolate ion currents flowing through Ca_V_2.2 (*N*-type) calcium channels. Ca_V_2.2 calcium channel current was defined as the component of the current sensitive to 100 μM Cd^2+^ in the presence of 5 μM nifedipine [12]. The bath solution contained (in mM) 160 NaCl, 2.5 KCl, 10 4-(2-hydroxyethyl)-1-piperazineethanesulfonic acid (HEPES), 8 Glucose, 5 CaCl_2_, 1 MgCl_2_ and 0.0002 tetrodotoxin (TTX). It was adjusted to pH 7.4 with NaOH. The internal solution contained (in mM) 140 CsCl, 20 tetraethylammonium chloride (TEA-Cl), 10 HEPES, 0.1 1,2-bis(2-aminophenoxy)ethane-*N*,*N*,*N*′,*N*′-tetraacetic acid (BAPTA4Cs), 5 MgCl_2_, 4 Mg_2_ATP, 0.3 Na_2_GTP and 0.1 leupeptin. This solution was adjusted to pH 7.2 with CsOH. For toxin application, peptide RsXXIVA was stored in a stock solution in distilled water at 30 μM and diluted to 0.03, 0.3, 3 and 24 μM in the external recording solution immediately prior use. The toxin was locally superfused by pressure injection using an Eppendorf 5246 transjector and a 5171 micromanipulator (Eppendorf, Madison, WI, USA) from a large borosilicate pipette (3 to 5 μm tip diameter) located 20 to 50 μm from the cell membrane. Injection pressure was set to 250 hPa, compensation pressure to 10 hPa and injection duration to 20 s. All chemicals were obtained from Sigma.

### 2.8. Current Measurements and Analysis

Membrane currents were measured by the whole-cell configuration of the patch-clamp technique [13], using an EPC-9 amplifier (HEKA Instruments) and borosilicate glass pipettes with a resistance of 1–2 MΩ when filled with the internal solution. Once the whole-cell configuration was established, the cells were held at −80 mV, capacity transients were cancelled and series resistance was compensated to >70%. Voltage protocols were generated and data were digitized and recorded using PULSE software (HEKA Instruments). Currents were typically low-pass-filtered at 3 kHz (3-pole Bessel filter) and were sampled at 10 kHz. Linear components were subtracted by the P/4 protocol from a holding potential of −80 mV. Since the magnitude of the Ca_V_2.2 current depended on cell size, current data are presented as a normalized current density. Only spherical cells of small diameter with no visible processes were selected for recordings. Where appropriate, data was recorded as the mean ± SEM Statistical significance was determined using the unpaired Student’s *t* test. Results were considered significant if *p* < 0.05.

### 2.9. Experimental Animals

Adult male imprinting control region (ICR) mice weighing 23 ± 2 g were maintained in cages with a 12/12-h light/dark cycle and constant room temperature (23 ± 2 °C), with standard laboratory food and water *ad libitum*. Animals where acclimatized at least 30 min before testing. Each animal was used only once. The protocols were approved by the ethics committee of the Instituto de Biotecnología of the Universidad Nacional Autónoma de México (UNAM), campus Morelos and were carried in accordance with the current guidelines for the care of laboratory animals and the ethical guidelines for investigation of experiments in conscious animals. The number of animals and intensity of noxious stimuli were the minimum necessary to demonstrate consistent effects of toxin and drug treatments.

### 2.10. Hot-Plate Test

A hot-plate test was used for measuring the potential anti-nociceptive effect of RsXXIVA to an acute thermal stimulus [14]. The animals were allowed to acclimate an hour before testing. Three animals per group were used; each group was intraperitoneally (IP) injected with either 0.85 mg/kg of RsXXIVA or a control solution (phosphate buffered saline (PBS) 1× or Nalbufin 4 mg/kg). Each animal was tested twice, 30 and 45 min post-injection. A single animal was placed in a plexiglass cylinder (20 × 25 cm) on the hot plate (Harvard Apparatus, Panlab model LE7406) maintained at 55 °C. The time taken for the animals to lick its paws or jump was registered immediately after placing the animal into the cylinder. The cutoff time was 30 s to avoid tissue damage. Nalbufin is an opioid analgesic drug, and it was used as a positive control, because it has less respiratory depression effects than other opioid drugs.

### 2.11. Formalin Test

The formalin test generates a biphasic pain-like behavior; that is, a phase 1 (acute pain), characterized for a short, but immediate response lasting the first 5 min after the hind paw injection (liking and biting of the injected paw); and a phase 2 (chronic pain), characterized by a prolonged response starting approximately 11 min after injection. The Dubuisson and Dennis procedure was used [15], with some modifications. Briefly, adult male ICR mice (23 ± 2 g) were used and acclimatized one hour before the test; food and water were available *ad libitum*. Animals were placed one at a time in a plexiglass chamber (29 × 22 × 14 cm); each chamber had mirrors on the three sides. Either the toxin (0.85 mg/kg) or control (PBS 1× as a negative control and Ketorolac 10 mg/kg and Tramadol 5 mg/kg as positive controls) was IP injected in a volume of 200 μL. Fifteen min after injection, 20 μL of 2.5% formalin were subcutaneously (SC) injected into the plantar right hind paw, and mice were immediately placed in the plexiglass chamber. Time in seconds of hind paw licking occurring in the first minute was counted, and the same was done in five min intervals, up to 45 min post-formalin injection. Phase 1 was defined as the first 5 min following formalin injection (0–5 min), and phase 2 was defined as 11–45 min post-formalin injection.

### 2.12. Statistical Analysis

Hot-plate and formalin test data were reported as the mean ± SEM. The anti-nociceptive effect of RsXXIVA was statistically compared with the controls by one-way ANOVA, followed by an unpaired *t* test (two-tail), where *p* < 0.05 was considered as significant. Statistical analyses were performed by using the GraphPad Prism software (GPW5-050878-RAF-4725). 

## 3. Results

### 3.1. Isolation and Sequence of the Native Peptide

**Figure 1:**
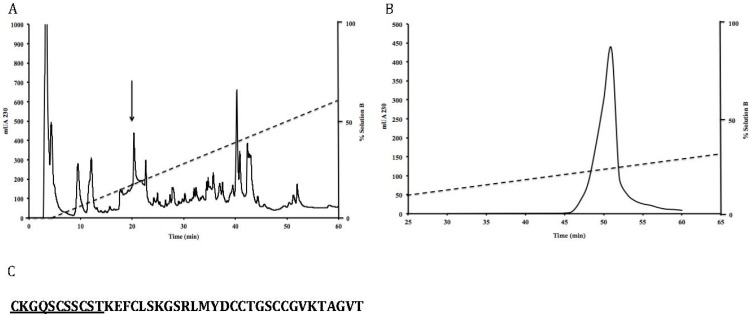
Purification of RsXXIVA by reversed-phase high-performance liquid chromatography (RP-HPLC). All purification protocols were conducted at room temperature; the elution was monitored for absorbance at 230 nm. Panel **A**: fractionation of the venom extract from *C. regularis* by an analytical C_18_ column eluted with a linear gradient from 0% to 60% of buffer B, over 60 min at 1 mL/min. Panel **B**: the peak indicated by the arrow in panel A was further purified on a micro-bore C_18_ column using a linear gradient from 10% to 30% of buffer B, over 60 min at 200 μL/min. Panel **C**: the amino acid sequence of RsXXIVA was determined by mass spectrometry, and the amino terminal sequence was confirmed by automated Edman degradation (underlined amino acids).

From the crude venom of *C. regularis*, a component of 4121.5 Da was identified and purified (Figure 1A,B). It represented 7% of the total venom components determined by its relative peak area compared to the total area of the HPLC fractions. After analysis by Edman degradation and liquid chromatography-mass spectrometry (LC-MS), its primary structure was determined (Figure 1C). This toxin has a new Cys framework, C-C-C-C-CC-CC, which has never been reported before. Based on the nomenclature of Olivera and Cruz [16], this conotoxin was named RsXXIVA, where “Rs” refers to the specie’s name, *C. regularis*, the number, “XXIV”, specifies the type of Cys framework (C-C-C-C-CC-CC) and “A” stands for the letter assigned to the first peptide isolated within this new cysteine framework.

**Figure 2:**
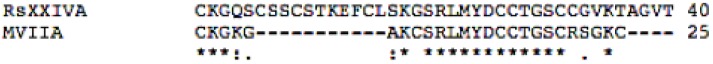
Sequence comparison. Comparison of the RsXXIVA and MVIIA conotoxins found in *C. regularis* and *C. magus*, respectively. Identical residues (*), conserved substitutions (:) and semi-conserved substitutions (.) among these conotoxins are shown along the bottom.

Considering the high identity at position 18 to 30 and 7 to 20 of RsXXIVA and MVIIA, respectively (Figure 2), it was decided to test the effect of RsXXIVA on Ca_V_2.2 calcium channels. Two relevant residues, Lys2 and Tyr13, which are the most important amino acids in MVIIA for binding to the calcium channels [17], are present in both toxins. The primary structure of RsXXIVA is quite different from MVIIA, since it has 40 amino acid residues, whereas MVIIA has only 25. However, amino acid sequence comparison, using CLUSTALW, showed that among the amino acids present in both structures (not including the corresponding sequences absent in ω-MVIIA) is about 64% identical. Only nine of the 25 amino acid residues of RsXXIVA, pairwise compared with MVIIA (in the corresponding positions of both sequences), are different. The segments are highly identical, corresponding to two loops of ω-MVIIA [18]. 

### 3.2. Effects of Toxin RsXXIVA on Ca_V_2.2 Calcium Channel Current

**Figure 3:**
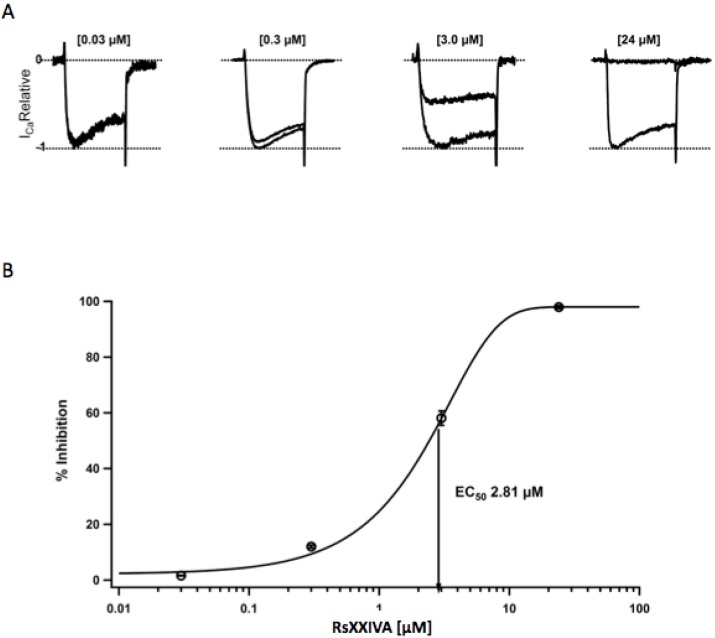
Dose-response relationship for calcium current inhibition by RsXXIVA from *C. regularis* venom. (**A**) Relative superimposed calcium current traces under control conditions and under RsXXIVA application at different concentrations are shown at the top of every current trace. Dotted lines correspond to the zero current and the maximum current. (**B**) Symbols represent the average percentage of current inhibition at each toxin concentration, plotted on a semi-logarithmic scale. Data were fitted to a sigmoid function (solid line), with the following equation: *y* = [(A_1_ − A_2_)/{1 + ([*Tx*]/[*Tx*]_0_)*^n^*}] + A_2_. The midpoint value, [*Tx*]_0_, was 2.8 μM, which corresponds to EC_50_, and the Hill coefficient calculated from this set of data was *n* = 0.94.

Since it is well known that MVIIA inhibits Ca_V_2.2 channels [19], it was decided to observe the effect of peptide RsXXIVA on the same type of ion-channel. Thus, the effect of RsXXIVA was assessed on neurons from the superior cervical ganglion, since the Ca_V_2.2 current represents >90% of the total calcium current in this preparation [20,21,22]. Ca_V_2.2 currents were elicited by 30 ms depolarization at −10 mV under whole cell configuration. RsXXIVA inhibited Ca_V_2.2 current accordingly with its similarity to MVIIA conotoxin. Figure 3A shows the superimposed relative calcium currents before and during application of different concentrations of RsXXIVA. As expected, Ca_V_2.2 current inhibition was dependent on the concentration of RsXXIVA. That is, when 0.03, 0.3, 3 and 24 μM of RsXXIVA was applied to neurons, calcium currents were inhibited by 1.6 ± 0.1, 12.0 ± 0.5, 58.1 ± 2.6 and 97.9 ± 0.5% (Figure 3A, *n* = 5), respectively. The dose-response data was fit by a single sigmoid curve with a value of EC_50_ equal to 2.8 μM (Figure 3B) and a Hill coefficient of 0.94, which suggests a molar ratio 1:1 of ligand/receptor.

This concentration is quite high, when compared to the values found for other conotoxins affecting ion-channels [23]. For this reason, we have decided to conduct experiments related to analgesia, as described below.

**Figure 4:**
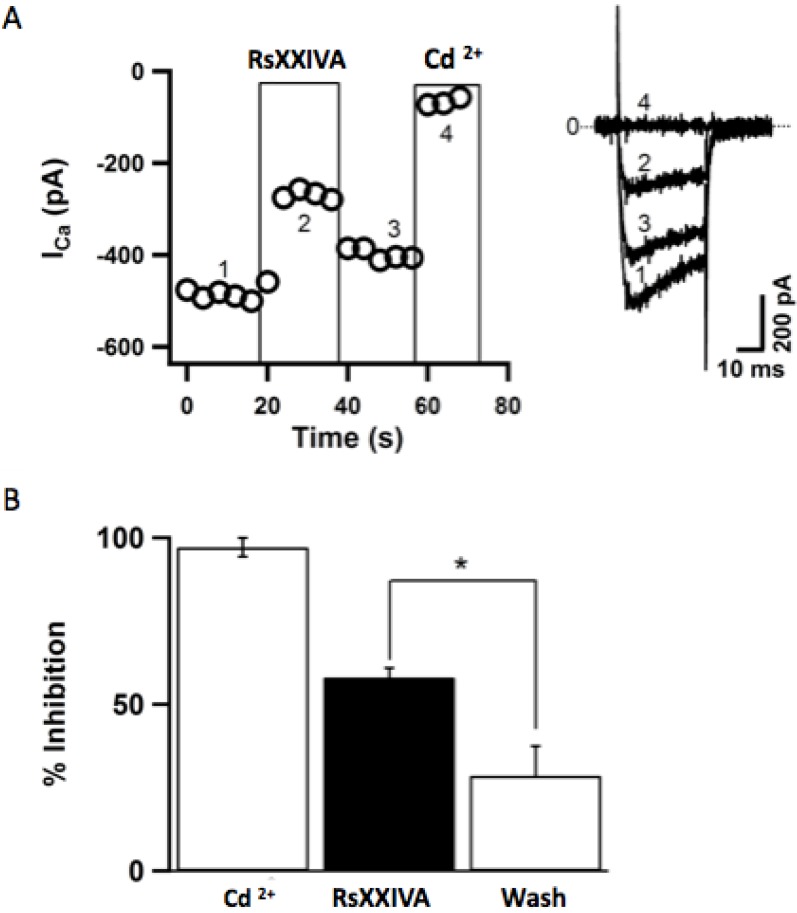
Time course of Ca_V_2.2 current inhibition by application of 3 μM of RsXXIVA in rat superior cervical ganglion (SCG) neurons. (**A**) Symbols are mean calcium currents of the test pulse before (1), during (2) and after (3) toxin application. The test pulse was delivered every 4 s, and at the end of the experiment, 100 μM CdCl_2_ was applied (4). The inset is representative of calcium currents for each condition. (**B**) The summary of inhibition and recovery (wash) of calcium current under toxin RsXXIVA application. * Represents *p* < 0.05.

Figure 4 shows the time course of the effect of 3 μM toxin RsXXIVA on Ca_V_2.2 currents. Here, toxin was applied for 20 s with a micro-perfusion system, as described in the Material and Methods section. Ca_V_2.2 currents were measured as the average of inward current between 4 and 5 ms elicited by the test pulse every 4 s before, during and after toxin application. RsXXIVA inhibited Ca_V_2.2 current in less than 4 s, and its effect was partially washed out, as can be seen in Figure 4A. After toxin washout, the current was restored quickly, from −442.9 ± 213.8 pA to −798.2 ± 313.4 pA (*n* = 5). Figure 4B summarizes the percentage of inhibition (58.1% ± 2.6%) and recovery (71.4% ± 8.8%) compared to the block with 100 μM Cd^2+^. These results show that the toxin RsXXIVA inhibits Ca_V_2.2 channels, despite its different cysteine framework, and it is partially reversible. Although the EC_50_ of RsXXIVA was high for inhibiting Ca_V_2.2 when compared to the EC_50_ values of other conotoxins [11], it was decided to conduct experiments related to analgesia.

### 3.3. Analgesic Activity of RsXXIVA

**Figure 5:**
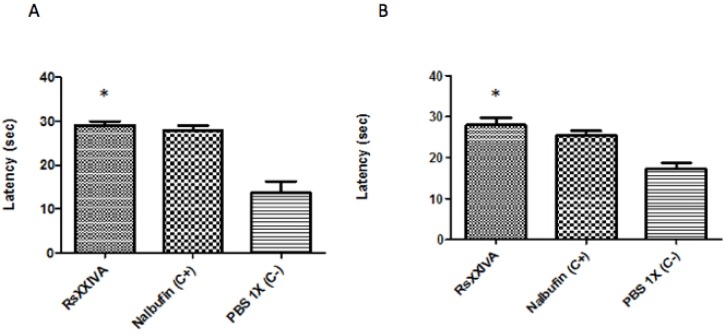
Effect of RsXXIVA (0.85 mg/kg intraperitoneal (IP)) in the hot-plate test. Adult male imprinting control region (ICR) mice where IP injected with 200 μL of either toxin or Nalbufin (4 mg/kg) as the positive control and PBS 1× as the negative control. Data are expressed as the mean ± SEM (*n* = 3). (**A**) The biological effect of RsXXIVA at 30 min post-injection. RsXXIVA shows (with 95% CI = 28.29 ± 3.35) an analgesic effect in acute thermal pain at 30 min post-injection in reference to the control group. (**B**) The biological effect of RsXXIVA 45 min post-injection. RsXXIVA shows an analgesic effect in acute thermal pain at 45 min post-injection (with 95% CI = 28.10 ± 3.71). Data were compare by one-way ANOVA, followed by Dunnett’s multiple comparison test * represents *p* < 0.05, considered as significant.

To test whether RsXXIVA has an effect on acute thermal pain, the hot-plate set-up system was used. Mice were placed on a hot plate at 55 °C until they started licking or lifting their hind paws as an indication of acute thermal pain. RsXXIVA, showed a similar effect as Nalbufin, which is a potent analgesic drug. The analgesic effect of RsXXIVA was detected after 30 (Figure 5A) and 45 (Figure 5B) min post injection, and in both cases, the analgesic effect was better than that of Nalbufin and significantly better than the control group (*p* < 0.05).

**Figure 6:**
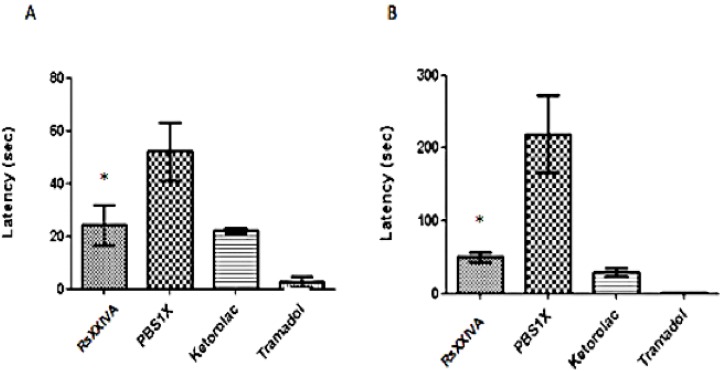
Effect of RsXXIVA (0.85 mg/kg IP) in formalin test. Adult male ICR mice where subcutaneously (SC) injected with 2.5% formalin (20 μL) 15 min before IP administration of 200 μL of either toxin or control Ketorolac (10 mg/kg) and Tramadol (5 mg/kg) as the positive control and PBS 1× as the negative control. Data are expressed as the mean ± SEM (*n* = 3). (**A**) The biological effect during phase 1 (0–5 min). RsXXIVA reduced the licking time compared with the control group (with 95% CI = 24.33 ± 14.69), reflecting activity in response to acute pain. (**B**) The biological effect during phase 2 (11–45 min). RsXXIVA appeared to have an anti-nociceptive effect compared with the control group (with 95% CI = 50.05 ± 13.33), which reflects chronic pain. Data were analyzed with one-way ANOVA, followed by Dunnett’s multiple comparison test. * Represents *p* < 0.05, considered as significant.

Also, we assessed the analgesic effect of RsXXIVA in a model of persistent inflammatory pain in mice using the formalin test. RsXXIVA significantly reduced the hind paw licking during both phase 1 (Figure 6A) and phase 2 (Figure 6B), compared with the control group. In accordance with the pain model, the acute pain phase 1 pain is caused by a direct effect on nociceptors, whereas chronic pain phase 2 pain is caused by chronic inflammatory responses [24]. As it can be seen, RsXXIVA had a similar effect as Ketorolac, which is not a steroidal drug to treat acute and chronic pain related to inflammatory events. 

## 4. Discussion

*Conus regularis* is a vermivorous cone snail species whose venom components have not been previously explored. This novel peptide, RsXXIVA, has a unique eight-cysteine pattern (C-C-C-C-CC-CC), which clearly diverges from other known conotoxins. Classical ω-conotoxins show highly conserved residues, such as Lys2 and Tyr13. Lys 2 is present amongst all ω-conotoxins, except MVIID and TVIA, and Tyr13 is unequivocally the single most important residue for binding *N*-type calcium ion channels [17]. Remarkably, RsXXIVA has equivalent residues to Lys2 and Tyr13 (Lys2 and Tyr24, Figure 2) [25]. The key residues (Lys and Tyr) appear in different position between ω-conotoxins and RsXXIVA, in loops 1 and 2 in ω-conotoxins and in loops 1 and 4 in RsXXIVA. On the other hand, these residues are flanked by the same amino acids in bout toxins. Further modeling or structural analysis certainly will clarify if these Lys and Tyr residues share the same structural positions in both RsXXIVA and MVIIA. 

Conotoxins containing four disulfide bonds have been relatively less explored. In fact, with the identification of RsXXIVA, only seven different peptides with four disulfide bonds have been reported [25,26,27,28,29,30]. The most extensively studied four disulfide bond conotoxin group is the I-superfamily group [31]. In fact, only the disulfide bond connectivity of the conotoxins ι-RXIA, belonging to this I-conotoxin group, has been determined [26]. 

The studies of ω-conotoxin MVIIA and its effectiveness as an analgesic drug favored is commercial application (Prialt, under FDA approval). However, serious side effects have been observed in its use. As a result, warnings regarding the unsuitability of this drug for use in patients with a pre-existing history of psychosis have been implement [32]. The on-going discovery of novel peptides with analgesic effects, acting on calcium channels, such as the ω-conotoxins, may provide the necessary insight for the development of new drugs targeting Ca^2+^ channels for pain treatment. This could increase drug potency and receptor targeting in humans, while minimizing their potentially adverse side effects. 

At present, there are few available drugs that target calcium channel receptors for pain treatment (*i.e.*, Prialt, Gabapentin and Pregabalin). Conversely, while several drugs that affect Ca^2+^ channels might be in development, there are few new voltage gated calcium channel (VGCC) blockers in clinical trials. Therefore, there is an urgent need for the development of new drugs targeting Ca^2+^ channels for pain treatment. Hereby, our results show that Ca_V_2.2 currents are inhibited by RsXXIVA toxin in a dose-response manner and a reversible manner. However, as already mentioned earlier, the affinity is not as high as expected, and it could well be that the exact target of RsXXIVA is not the Ca_V_2.2 ion channel. For this reason, experiments showing analgesic effects were conducted. In fact, our results support the conclusion that RsXXIVA has an anti-nociceptive effect based on the hot-plate and formalin tests. This is in good agreement with the blockade of nociceptive neurotransmission by altering Ca_V_2.2 channel function or any of the other channels described to be involved in analgesia. The high density of Ca_V_2.2 channels at the ganglionar level and presynaptic terminals is well-documented [33], and for this reason, these channels could be used as a molecular target for pain *in vitro* experiments. In our model, SCG neurons are the most used native preparation to assess Ca_V_2.2 (*N*-type) channel function, due to their richness of these receptors (>90%). The remaining Ca^2+^ channels (5%–10%) were readily eliminated by nifedipine. In our hands, toxin was applied to SCG cultured cells at a relatively high concentration (3 μM), a value near the EC_50_, by means of a high-precision micro-perfusion system. In contrast, previous studies indicated that MVIIA was markedly more potent (IC_50_ 32 nM) when assayed in a similar model [34]. This different rank-order of activity could be supported with the net charge distribution in both conotoxins, which is an important factor that may affect the binding potencies. The net charge for MVIIA is +5 and for RsXXIVA is +2.6 It has been considered that there is a threshold net charge for the binding of a toxin to the target, for example, loss of any single charged residue in ω-conotoxin GVIA (net charge of +5) results in a drop in potency as the net charge is reduced to only +4 [35].

The fast installation of the inhibition of the Ca_V_2.2 channel current induced by RsXXIVA indicates a direct action on the receptors. Unexpectedly, the inhibition of the Ca_V_2.2 channel current occurred reversibly. This is also in favor of the rapid access of the toxin to the binding site on the channel molecule. Again, the channel pore is a feasible candidate for toxin action. Direct VGCC blockade or modulation will always have a place in the treatment of neuropathic pain.

Further investigation should be made, however, to clarify the mechanistic action of RsXXIVA toxin, which is beyond the scope of this communication. Whatever the mechanism turns out to be, the inhibition observed on the Ca_V_2.2 channel current, along with the anti-nociceptive actions supported by the pain tests, justifies the use and possible relevance of RsXXIVA in future investigations related to pain control. Most toxins that blocks Ca_V_2.2 channels, do not have anti-nociceptive action when IP administration is used; this is not the situation for RsXXIVA. Even when RsXXIVA is able to block Ca_V_2.2 channels, it seems unlikely that these Ca_V_2.2 channels could be the targets for the anti-nociceptive effect of this toxin *in vivo*. Further assays should be done on peripheral nociceptive targets, such as the Transient Receptor Potential channel. 

## 5. Conclusions

This manuscript describes the isolation and characterization of a new peptide (RsXXIVA) from the venomous duct of the vermivorous marine cone snail *Conus regularis*, collected along the Pacific coast of Mexico. The peptide shows a novel cysteine disulfide pairing and a segment composed of 14 amino acid residues identical to that of the analgesic peptide MVIIA (known as Prialt) from *C. magus*. RsXXIVA shows nociceptive properties suggesting that it might be implicated in Ca_V_ ion channels function. These results constitute an important piece of information for future design of analgesic peptides based on residue modifications in specific loops of proteins associated to nociception.
